# Knowledge and practice of radiation protection in the operating theater among orthopedic surgeons

**DOI:** 10.1117/1.JMI.9.6.066002

**Published:** 2022-11-11

**Authors:** Badera Al Mohammad, Monther Gharaibeh, Maram Al Alakhras

**Affiliations:** a Jordan University of Science and Technology, Faculty of Applied Medical Sciences, Allied Medical Sciences Department, Irbid, Jordan; b The Hashemite University, Faculty of Medicine, Department of Special Surgery, Zarqa, Jordan

**Keywords:** radiation protection, medical imaging, orthopedic surgeons, orthopedics

## Abstract

**Purpose:**

Many orthopedic procedures, particularly minimally invasive surgeries that require fluoroscopic imaging, present a radiation exposure risk to the orthopedic surgeon. Surgeons may have a higher risk of developing cancer if they receive significant amounts of radiation. Using personal protective equipment (PPE) and appropriate imaging device positioning, plays an important role in reducing the surgeon’s radiation exposure. However, there is a lack of knowledge about the surgeon’s radiation safety awareness and practices. Therefore, the aim of this study is to investigate the practices and radiation protection knowledge of orthopedic surgeons in the operating theater.

**Approach:**

A nationwide survey was conducted from October 2021 to January 2022 to evaluate the radiation protection practices and awareness of orthopedic surgeons in Jordan. Normalized practice and knowledge scores were evaluated through the survey and compared between different groups. Descriptive statistics were used to present the surgeon’s practices and radiation protection knowledge. Student’s t-test was used to compare the outcomes between surgeons that received radiation protection training and surgeons who did not. Using ANOVA analysis, we compared the score outcomes for all the other variables.

**Results:**

The surgeons that received radiation protection training had significantly higher practice score 39.6% compared with 31% for the group that did not have training (p=0.01). No statistically significant difference in the knowledge scores was found between the two groups. Although 91% of the surgeons reported using some kind of PPE, only 5.5% used a dosimeter badge during surgeries.

**Conclusion:**

There is an obvious deficit in radiation safety training of orthopedic surgeons.

## 1 Introduction

Advancements in radiation technology have led to a widespread of its use in medicine. Using fluoroscopy in the operating theater has the benefit of providing the orthopedic surgeon with the ability to perform minimally invasive surgeries and improved visualization of the internal structures. This has resulted in fewer surgical complications when compared to open surgeries.^1^^,^^2^ However, the increase in the use of the fluoroscopically guided procedures during surgeries, has led to a rise in ionizing radiation exposure to orthopedic surgeons.^1^ Research has shown that orthopedic surgeons are in the top three most exposed medical professionals to ionizing radiation.^3^ During the fluoroscopy procedures, the orthopedic surgeon may receive significant amounts of radiation to their hands from the primary beam and to the rest of their body from scattered radiation.^4^ A number of studies have reported a higher risk of developing different types of cancers among orthopedic surgeons due to radiation exposure.^5^^–^^7^ In addition to cancer, cumulative radiation exposure has demonstrated an increased risk of developing cataracts.^4^^,^^8^^,^^9^

Identifying the occupational practices of fluoroscopy use and radiation protection knowledge of orthopedic surgeons is of paramount importance when developing strategies that will help reduce radiation exposure and hence its harmful effects.

Therefore, the aim of this study is to investigate the practices and radiation protection knowledge of orthopedic surgeons in Jordan.

## 2 Methodology

A cross sectional study was conducted after receiving the appropriate ethical approval at our institution. The data were collected from October 2021 to January 2022 to evaluate the radiation protection practices and awareness of orthopedic surgeons in Jordan. Around 470 orthopedic surgeons actively working in Jordan were primarily contacted for this survey by phone to inquire about their willingness to participate through the Jordanian Orthopedic Association. The surgeons that chose to participate were either sent an email containing the online survey or were visited by research assistants in person at their place of work to help conduct the survey on a digital tablet device. Orthopedic residents and specialists, working in university, public, military, and private hospitals were included in the study.

After reviewing a number of studies^10^^–^^13^ that investigated radiation safety measures of medical personnel in the operating theater using a survey, we formulated our questionnaire. Moreover, the questionnaire was evaluated by consultation with a radiation protection university professor, a consultant orthopedic surgeon, and a radiologist. The questionnaire covered a range of topics; the first part focused on collecting the participants’ demographic data. The second examined how frequently the surgeons are exposed to radiation in the operating theater. The third part focused on which safety measures are applied during operations and whether the participants have had any radiation protection training. The last part was investigating their knowledge on specific radiation protection principles and radiation exposure harms. Participation in this study was completely voluntary and the participants signed written consent forms about their willingness to take part in the study.

The data from the completed questionnaires were collected on Excel Spreadsheet and analyzed using Statistical Package for the Social Sciences (SPSS) file (version 20.0).

### 2.1 Knowledge Score

Seven questions were used to evaluate the surgeon’s knowledge of how to reduce their and the patient’s radiation exposure using the knowledge score. Each correct answer received a point and the end scores were normalized to 100. Table 1 demonstrates the questions, the choices, and the correct answer.

**Table 1:** Knowledge questions used for knowledge score assessment.

Knowledge questions
1. In your opinion, which part of the surgeon’s body has the greatest exposure to direct radiation during surgical procedures?
a. Eyes
b. Thyroid
c. **Hands**
d. Gonads
e. Feet
2. If the distance between you and the x-ray tube increased from 1 to 2 m, in your opinion, how much will the radiation dose decrease?
a. No decrease in radiation exposure
b. The radiation exposure will reduce to half
c. **The radiation dose will reduce to a quarter**
d. The radiation dose will reduce to one eighth
3. To reduce the radiation exposure level to the surgeon, the x-ray tube should be positioned:
a. **Under the patient (under couch)**
b. Above the patient (over couch)
c. There is no difference
4. In the lateral C-arm imaging, standing on which side of the table from the x-ray tube can greatly decrease radiation exposure?
a. Same side as the x-ray tube
b. **Opposite side from the x-ray tube**
c. There is no difference
5. How does using magnification while imaging affects the radiation dose to the surgeon and the patient?
a. **Increase**
b. Decrease
c. There is no difference
6. Does using collimation (decrease the area of the direct x-ray beam on the patient) reduce radiation exposure?
a. **Yes**
b. No
7. In order to reduce further the radiation exposure to the patient, which of the following is applied?
a. **The image intensifier is kept close to the patient**
b. The image intensifier is kept far from the patient

Correct answers are written in bold.^14^

### 2.2 Practice Score

Evaluating which practices the surgeon applied to reduce the radiation exposure to themselves and the patient was performed by using five questions with a total score out of 7 (Table 2). One question regarding which radiation protection equipment does the surgeon use had a maximum of three points; if the answer was none, they received zero points, using only one of the following (long lead apron, lead vest, thyroid shield, lead goggles, lead gloves, or gonadal shield) received one point, if they were using two different equipment they will obtain two points and three points were given if they used three different types of equipment or a mobile radiation protection screen. Here, as in the knowledge score, the end scores were normalized to 100.

**Table 2:** Demonstrates the questions used to evaluate the surgeon’s practice scores.

Practice questions
8. Which radiation protection equipment do you use? (choose more than one if applicable)
a. None
b. Lead apron (long)
c. Lead vest
d. Thyroid shield
e. Lead glasses
f. Lead gloves
g. Gonad shield
h. Mobile radiation protection screen
9. How often do you use the radiation protective equipment?
a. Always
b. Most of the times
c. Half of the times
d. Sometimes
e. Never
10. Do you use a dosimeter badge during the operations (dosimeter is a device used to measure the absorbed dose of radiation)
a. Yes
b. No
11. How far away from the x-ray device do you stand during fluoroscopy use?
a. I don’t change my place
b. 1 to 2 steps away
c. 1 m away
d. 2 m away
e. More than 2 m away
12. During the surgery, when radiation exposure is used, does the patient wear any radiation protection equipment such as lead apron, thyroid shield, gonadal shield, etc…?
a. Yes
b. No

### 2.3 Statistical Analysis

In this study, we will use descriptive statistics to present the surgeon’s practices and radiation protection knowledge in the operating theater. Student’s t-test will be used to compare the score outcomes between two groups; orthopedic surgeons who had received radiation protection training and surgeons who did not receive any training. Using ANOVA analysis, we compared the score outcomes for all the other variables. Significance level was set at (p<0.05)

## 3 Results

**Fig. 1:**
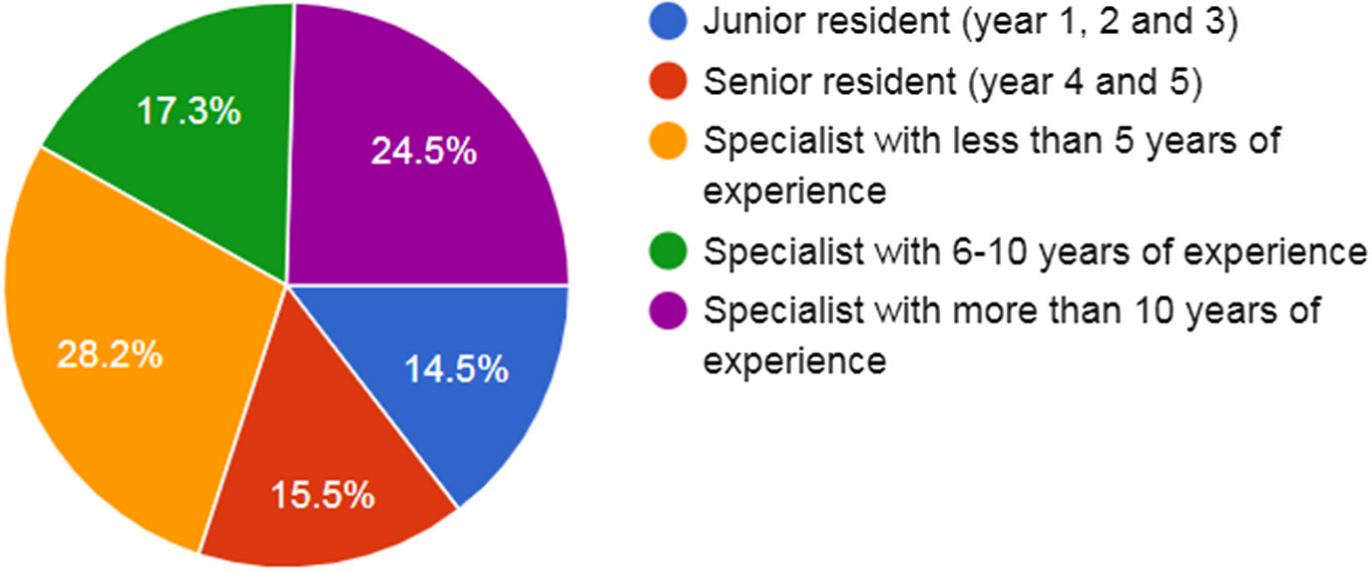
Years of experience in orthopedics.

A total of 114 orthopedic surgeons participated in the study; four participants were excluded because their questionnaires were not filled completely (response rate 23.4%). Sixteen surgeons (14.8%) worked in university, 43 (39.8%) in public, 25 (23.1%) in military, and 24 (22.2%) in private hospitals. Figure 1 demonstrates the years of experience in orthopedic surgery.

Half of the orthopedic surgeons (55) performed more than four surgeries per week that required the use of fluoroscopic radiation, around 35% (38) had an average of two to three surgeries per week and the rest performed surgeries once a week.

One-third, 33.6% (37) of all the participants had not received any type of radiation protection training during residency or afterward.

**Fig. 2:**
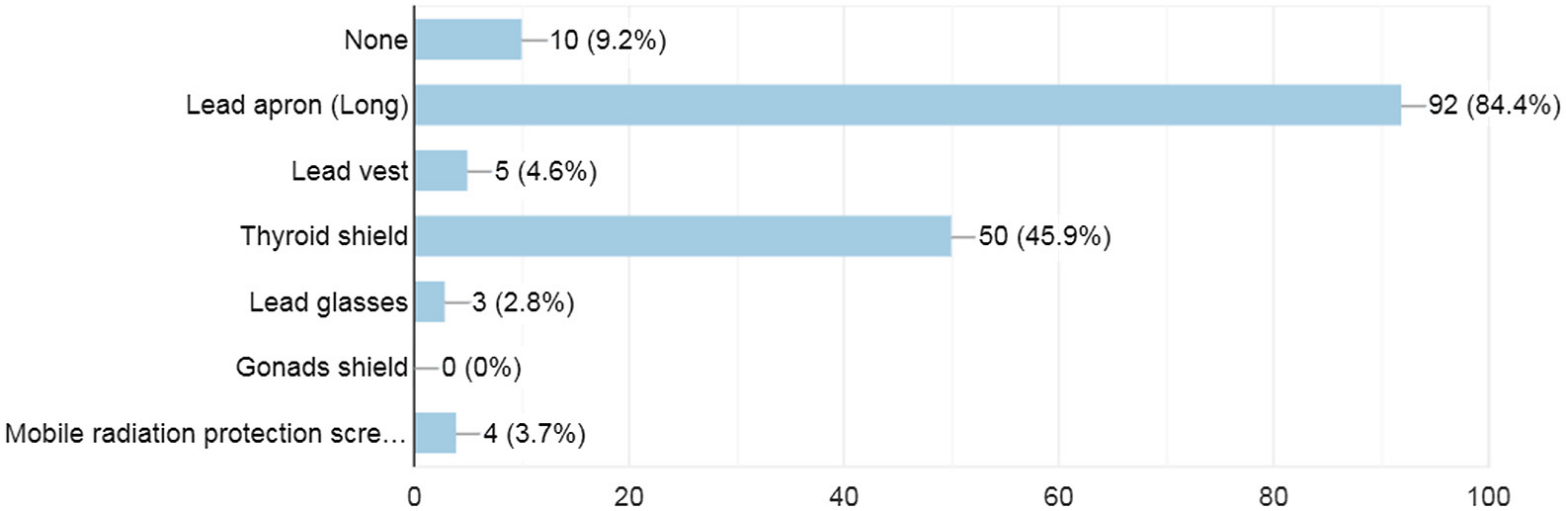
Types and frequencies of radiation protection equipment used in orthopedic surgeries.

Among the 110 orthopedic surgeons enrolled in the study, around 91% reported using some kind of radiation personal protective equipment (PPE), with lead apron 84% (92) being the most commonly used PPE type and lead glasses 2.8% (3) being the least common type used. Furthermore, only 64% (70) reported using the PPE always or most of the time, whereas the rest reported using it less than that. Figure 2 summarizes the types of radiation protection equipment used in the orthopedic surgeries and their frequencies.

Only 5.5% of the participants (6) used a dosimeter badge during surgeries and had the dosimeter readings sent routinely for measurements.

When asked about the inverse square law, only 29% (32) of the participants knew that the dose will be reduced to one quarter. Furthermore, just 62% (68) of the surgeons reported placing the x-ray tube under the patient’s table to reduce scatter radiation to the surgeon. Similarly, 43% (47) of the participants had knowledge that standing on the same side of the x-ray tube will decrease their radiation exposure.

Regarding reducing patient radiation dose during surgeries; 79% (87) of the surgeons reported not using collimation, whereas 87% (96) stated that the patient does not wear any type of radiation protection equipment.

### 3.1 Knowledge and Practice scores

When evaluating the knowledge score, no statistically significant difference was found between the group that received some type of radiation protection training 23.3%, and the group that did not 20.1% (p=0.2). On the other hand, when calculating the practice score, the surgeons that received radiation protection training had a significantly higher practice score of 39.6% compared to 31% for the group that did not have radiation protection training (p=0.01) (Table 3). There was no statistically significant difference in the practice or knowledge scores between orthopedic surgeons with different levels of experience or different practice settings (Table 4).

**Table 3:** Comparison between orthopedic surgeons that received radiation protection training and surgeons that did not.

Training scores	Knowledge scores			Practice scores		
	Mean	SD	P value	Mean	SD	P value
Received training	23.3	3.9	0.2	39.6	14.3	0.01
No training	20.1	2.4		31.0	17.1	

**Table 4:** Practice and knowledge scores compared with different levels of experience and practice setting.

		Practice score		Knowledge score	
		Mean (SD)	P value	Mean (SD)	P value
Level of experience	Junior resident	33.0 (15.4)	0.9	35.7 (19.5)	0.15
	Senior resident	30.4 (14.6)		46.4 (20.5)	
	Specialist (<5 y)	33.6 (14.5)		51.6 (18.3)	
	Specialist (6 to 10 y)	38.9 (17.7)		46.0 (25.3)	
	Specialist (>10 y)	33.7 (20.6)		41.7 (21.8)	
Practice setting	University	28.6 (21.5)	0.3	45.5 (29.1)	0.6
	Public	33.7 (16.3)		44.9 (19.1)	
	Military	32.7 (14.3)		49.4 (18.9)	
	Private	38.7 (15.5)		41.1 (21.6)	

## 4 Discussion

Since the discovery of radiation, its implementation in medical imaging has provided an enormous benefit for pathology diagnosis, however, its recurrent application has also increased the potential harm to the medical staff and the patient. Diagnostic radiation could cause up to 3% of future malignant masses diagnosed annually as reported by one review study.^15^

In orthopedic surgery, the use of medical radiation has been a crucial part of the practice. Furthermore, the introduction of minimally invasive orthopedic procedures has dramatically increased the use of the intraoperative fluoroscopic imaging. This in turn has resulted in exposing the surgeon to higher amounts of ionizing radiation.^4^ Furthermore, it has been demonstrated that orthopedic surgeons have an increased incidence risk of developing cancer when compared to healthcare workers that were not exposed to radiation.^7^

Decreasing direct radiation and wearing PPE reduces the exposure and its possible harmful effects on the surgeon. Moreover, understanding the scatter radiation direction may additionally reduce radiation exposure, particularly because the main radiation that the orthopedic surgeons are exposed to is scattered radiation.^16^ A number of studies have shown that the position of the surgeon and the radiation device can reduce scatter radiation to the surgeon.^14^^,^^17^^,^^18^ In our study, when asked about the proper position of the x-ray tube, only 63% of the surgeons knew that in order to reduce the scatter radiation to their radiosensitive organs, the x-ray tube should be placed under the patient’s table. Similarly, less than half of the surgeons had knowledge that, in lateral imaging, they should be standing on the opposite side of the x-ray tube since the highest rate of scatter is produced between the x-ray tube and the patient.^14^ The correct response rate is comparable to a study conducted in the UK by Raza et al.^19^ in which 55% of the orthopedic surgeons answered correctly on a similar question. Training the surgeons on the correct position of the x-ray tube is essential in reducing their radiation exposure.

Regarding their knowledge about the inverse square law, (intensity of the x-ray $\text{beam}=1/d^{2}$, d being the distance from the x-ray source), only about one-third of the participants answered the question correctly. The US National Council on Radiation Protection and Measurements recommends that the surgeon stands at least 2 m away from the radiation source.^16^

Our study assessed which radiation protective equipment orthopedic surgeons used during surgeries and how often they were worn. The results demonstrated that 91% of surgeons used some type of PPE, with the long lead apron being the most commonly used type (84.4%). Interestingly, only 2.8% and 46% of the surgeons in our study reported wearing lead glasses and thyroid shield, respectively, despite 41.3% and 86% of the participants correctly identifying the eyes and thyroid as one of the most sensitive organs to radiation. This may be due to the unavailability of these types of protective equipment, in most operating rooms. Similar trends of radiation protective equipment were described by Tuncer et al. in which 85% of the orthopedic surgeons reported using lead apron, 5% lead glasses, and a higher percent of 70% using thyroid shield.^20^ Raza et al.^19^ also reported a poor use of the thyroid shield and lead glasses in their study. Chronic radiation exposure has shown to have a damaging effect on the eye in the form of cataracts,^9^ however, wearing lead glasses will decrease radiation exposure to the eye by 90%.^21^ In addition, 85% of papillary carcinomas of the thyroid are considered to be radiation-induced^14^^,^^22^^,^^23^ and by using thyroid shield there is ∼50% reduction in the total exposure.^22^

The rates of use of the PPE in our study were comparable to the rates reported by similar studies.^12^^,^^24^ One such study was conducted during an international AO orthopedic surgery training course in Switzerland that involved orthopedic surgeons from North America, South America, Europe, Asia-Pacific, Africa, and the Middle East.^24^ They reported that the rates of using lead aprons, lead glasses, and thyroid shield were 70%, 3%, and 35.5%, respectively.

According to our study, only 5.5% of the orthopedic surgeons reported wearing a dosimeter, which is significantly lower than what has been reported by similar studies: 21%, 20%, and 47.3% in three different studies.^10^^,^^11^^,^^24^ This could be attributed to the lack of policies that enforce the use of dosimeters and that these devices are not readily available in all hospitals. The use of dosimeter and its regular measuring would accurately evaluate the radiation exposure to surgeons and correlate the measured dose with the threshold guidelines. As a consequence, exceeding radiation dose limits will be prevented.

One of the more important findings of this study is that only one-third of the participants reported receiving some type of radiation protection training. In contrast, a nationwide survey conducted in the United States reported that almost 80% of orthopedic residents received a general radiation safety training.^25^ In addition, our data demonstrated that the surgeons did not have adequate radiation protection knowledge as reflected by their relatively low knowledge and practice scores. The reason for this may be that currently there are no mandatory radiation protection courses that the orthopedic surgeons must undertake during their residency training.^26^ Most orthopedic surgeons that receive some type of radiation safety training do so in either conferences for continuing medical education points or optional trauma workshops. Introducing radiation usage policies and guidelines may be of a benefit for surgeons in the operating theater. Furthermore, improving radiation safety training courses can reduce scatter radiation to surgeons and decrease unnecessary radiation dose to the patients.

A few studies conducted worldwide have investigated other healthcare personnel^13^^,^^27^^,^^28^ and orthopedic surgeons’^20^^,^^29^^,^^30^ knowledge and practices regarding radiation safety in the operating theater. One such survey study investigated the responses of 1024 orthopedic surgeons and although they concluded that the surgeons had an inadequate knowledge in radiation protection their survey mainly investigated the surgeons practice and contained only one question that tested their radiation knowledge.^20^ However, we believe our study represents a more comprehensive survey that assesses multiple aspects of radiation protection knowledge and practices and provides objective and subjective data to describe the current findings.

The main limitation of our study is the low number of participating surgeons; larger sample size may have shown a statistically significant difference in the knowledge scores between surgeons that have received radiation protection training and the ones that have not. However, the response rate in our study of 23.4% versus 5% in a similar study is considered higher, thus more representative of the population.^29^ Additionally, according to our knowledge, this is the first study that investigates occupational radiation exposure to orthopedic surgeons in Jordan. Another limitation may be the self-selection bias, where the surgeons that responded to our questionnaire might be the ones who wanted to report that they did not have adequate radiation protection training.

## 5 Conclusion

Our findings indicate that there may be an obvious deficit in radiation safety training of orthopedic surgeons, as demonstrated by the falsely answered questions and the low knowledge scores in general. This signifies the need for a more thorough radiation protection training addressing important issues illustrated in the present study.

Furthermore, the unavailability of some types of PPE in the operating theater and the severe lack of use of personal dosimeter highlights the critical need to make this equipment more available to surgeons in order to reduce their occupational radiation exposure.
